# Identification of Beak and Feather Disease Virus in an Unusual Novel Host (*Merops ornatus*) Using Nested PCR

**DOI:** 10.1128/genomeA.01376-16

**Published:** 2016-12-08

**Authors:** Subir Sarker, Shubhagata Das, Karla Helbig, Shane R. Raidal

**Affiliations:** a Department of Physiology, Anatomy and Microbiology, School of Life Sciences, La Trobe University, Melbourne, Victoria, Australia; b School of Animal and Veterinary Sciences, Faculty of Science, Charles Sturt University, Wagga Wagga, New South Wales, Australia

## Abstract

The complete genome sequence of beak and feather disease virus (BFDV) was discovered from a rainbow bee-eater (*Merops ornatus*), a species of *Coraciiformes*. The genome consisted of 1,996 bp encoding two major bidirectional transcribed open reading frames. This is the first evidence of BFDV infection and complete genome characterization for this novel host species.

## GENOME ANNOUNCEMENT

Circoviruses are among the smallest and simplest of all known autonomously replicating viruses with a nonenveloped, icosahedral, and single-stranded circular DNA (ssDNA) genome of approximately 2 kb (1). Circoviruses sequences have been identified in numerous bird species, including parrots, pigeons, gulls, ducks, geese, swans, ravens, canaries, finches, starlings, and chickens (2). To date, a wide range of psittacine species both in wild and captive populations have been documented to be infected with beak and feather disease virus (BFDV) (3–9). However, the disease status of one of the Australian migratory birds, the rainbow bee-eater, which is regularly moving to and from Asia through the Terries Strait Islands, is unknown. It has been noted that members of the family *Meropidae* (commonly bee-eaters) have had an extremely rare chance of receiving avian influenza (10). Here, we report the identification and molecular characterization of a BFDV genome in rainbow bee-eater (*Merops ornatus*), a species of *Coraciiformes* unrelated to parrots and not previously known to be susceptible to any avian circovirus.

Dried blood spots collected from a juvenile rainbow bee-eater (sample ID, 14-1186/01; year of sampling, 2014; GPS location, −23.420757°S 133.5250201°E) were used as a source of genomic DNA, and extraction of DNA was carried out using established protocols (11). Published circovirus genome sequences were retrieved from the literature and NCBI GenBank (12), and two sets of degenerate primers were designed. The nested PCR was standardized and used for the amplification of a segment of a circovirus sequence described by Sarker et al. (13). Direct Sanger sequencing of the purified gel bands resulted in a 350-bp sequence after trimming off primer sequences, and a BLASTn search with these sequences returned multiple hits to the BFDV replication-associated gene. Consequently, additional standardized PCR protocol was used for the amplification and sequencing of the remainder of the genome of BFDV (4, 6, 13).

A preliminary BLASTn (14) analysis of the assembled sequence showed a significant match to BFDV. Further analysis of the complete genome of BFDV (1,996 nucleotides) from *Merops ornatus* revealed an overall 82.2 to 95.6% pairwise identity with other BFDV genomes available in GenBank, and the highest similarity (95.6%) to a BFDV from a wild red-tailed black cockatoo from Western Australia (KF385399) (6). The genome has the same basic structure as other BFDV genomes, which includes two major open reading frames (ORFs): ORF1 on the virion strand, encoding a replication-associated protein (Rep), and ORF2, encoding the capsid protein (Cap). Consequently, based on BLASTn (14) and BLASTp (15) analyses, ORF2 was more diverse than ORF1.

This study highlights the evidence of the rainbow bee-eater being an unusual host for BFDV infection. Some aspects of this case are difficult to explain fully without conducting ethically debatable experimental virus-transmission experiments. The rainbow bee-eater is a migratory bird that does not intimately share any ecological niche with any known parrot species. Therefore, this study may provide a unique opportunity for the better understanding of BFDV infectivity in the ecosystem.

### Accession number(s).

This whole-genome sequence of BFDV has been deposited in DDBJ/ENA/GenBank under the accession number KM823541. The version described in this paper is the first version.
